# The COVID-19 Pandemic during the Time of the Diabetes Pandemic: Likely Fraternal Twins?

**DOI:** 10.3390/pathogens9050389

**Published:** 2020-05-19

**Authors:** Shelley A. Cole, Hugo A. Laviada-Molina, Jeannette M. Serres-Perales, Ernesto Rodriguez-Ayala, Raul A. Bastarrachea

**Affiliations:** 1 Population Health Program, Texas Biomedical Research Institute and Southwest National Primate Research Center (SNPRC), San Antonio, TX 78227-0549, USA; scole@txbiomed.org; 2 Escuela de Ciencias de la Salud, Universidad Marista de Mérida, Mérida 97300, Mexico; halm611031@hotmail.com (H.A.L.-M.); jgmserres2014@hotmail.com (J.M.S.-P.); 3 Centro de Investigación en Ciencias de la Salud (CICSA), Facultad de Ciencias de la Salud, Universidad Anáhuac Norte, Naucalpan de Juárez 52786, Mexico; Ernesto.rodriguez@anahuac.mx

**Keywords:** COVID-19, diabetes, inflammation, adipose tissue dysfunction, cytokine storm, GEMM family study

## Abstract

An altered immune response to pathogens has been suggested to explain increased susceptibility to infectious diseases in patients with diabetes. Recent evidence has documented several immunometabolic pathways in patients with diabetes directly related to the COVID-19 infection. This also seems to be the case for prediabetic subjects with proinflammatory insulin resistance syndrome accompanied with prothrombotic hyperinsulinemic and dysglycemic states. Patients with frank hyperglycemia, dysglycemia and/or hyperinsulinemia develop systemic immunometabolic inflammation with higher levels of circulating cytokines. This deleterious scenario has been proposed as the underlying mechanism enhancing a cytokine storm-like hyperinflammatory state in diabetics infected with severe COVID-19 triggering multi-organ failure. Compared with moderately affected COVID-19 patients, diabetes was found to be highly prevalent among severely affected patients suggesting that this non-communicable disease should be considered as a risk factor for adverse outcomes. The COVID-19 pandemic mirrors with the diabetes pandemic in many pathobiological aspects. Our interest is to emphasize the ties between the immunoinflammatory mechanisms that underlie the morbidity and lethality when COVID-19 meets diabetes. This review brings attention to two pathologies of highly complex, multifactorial, developmental and environmentally dependent manifestations of critical importance to human survival. Extreme caution should be taken with diabetics with suspected symptoms of COVID-19 infection.

## 1. Introduction

**Figure 1:**
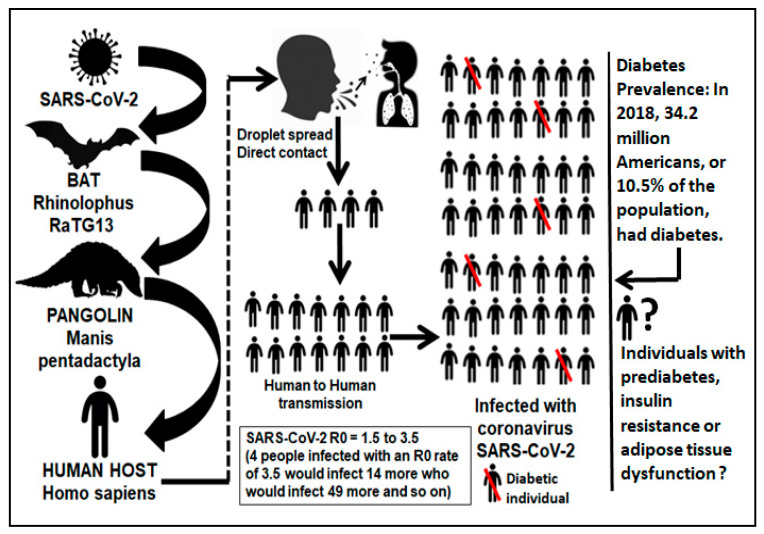
Human transmission of SARS-CoV-2. Five out of 49 COVID-19 infected individuals would be diabetic in the US. There is a lack of routine clinical criteria for the diagnosis of individuals with insulin resistance or adipose tissue dysfunction. These entities are accompanied by chronic low-grade subclinical inflammation perhaps acting as the underlying mechanism enhancing a cytokine storm hyperinflammatory state that triggers multiorgan failure in COVID-19 infected individuals.

In the last few months, rapid publications reporting on the outbreak of the novel betacoronavirus SARS-Cov-2 have flooded the scientific literature [1]. Soon after the number of cases outside China increased 13-fold and the number of countries with cases increased threefold, the World Health Organization (WHO) declared the novel coronavirus (COVID-19) outbreak a global pandemic (3/11/2020) [2]. The COVID-19 virus was first named 2019 novel corona virus (2019-nCoV)2 immediately after it was identified and isolated. Subsequently, the WHO officially changed its name to severe acute respiratory syndrome coronavirus 2 (SARS-CoV-2) [3]. This novel coronavirus is structurally related to the virus that causes severe acute respiratory syndrome (SARS) [4]. Previous to the current COVID-19 pandemic, there were reports that hyperglycemia and overt diabetes were considered independent predictors for higher mortality among patients with severe SARS vs. non-SARS pneumonia [5]. The Centers for Disease Control and Prevention (CDC) have also highlighted that patients with overt diabetes and/or the metabolic syndrome might have up to ten-times greater risk of death when they contract COVID-19 [5]. On average, each infected person spreads the infection to additional persons and the virus stays in the oropharynx early in the course of disease leading to a higher infectivity during presymptomatic stages (Figure 1). Sources from the MRC Centre for Global Infectious Disease Analysis at the Imperial College London have stated that minor differences in transmission rates (R0, infection basic reproduction number) for viruses can lead to drastic differences in the final number of overall infections. They have mathematically estimated that the COVID-19 virus has an R0 of 1.5–3.5 meaning that if four individuals were infected with the virus with an R0 of 3.5, they would infect 14 more who would infect 49 more and so on (Figure 1). A mean interval of 9.1–12.5 days between the onset of illness and hospitalization has been documented [6]. Very early reports from the city of Wuhan in Hubei province, China, indicated that the patient mean age was 59 years old, 56% of affected were males and, as previously observed with influenza, a higher mortality was found among patients with preexisting comorbidities and the elderly [7]. Diabetes stood out among those coexisting conditions [8,9].

Although diabetes is classified as a non-communicable disease [10], evidence indicates a major role of infectious agents as possible interactive factors associated with diabetic status [11]. Common infections occur more frequently in diabetic individuals. Moreover, infectious agents have a possible immunologic impact on different classes of diabetes (Type 1 or 2), accompanied by the diabetic genetic component and combined with immune dysfunction [12]. This review centers on the presence of diabetes as a potential risk factor for severity and death for a subgroup of patients with severe COVID-19 disease and dysglycemia requiring hospitalization, oxygen support and admission to an intensive care unit. It also includes prediabetic states such as the insulin resistance (metabolic) syndrome and adipose tissue dysfunction and its probable impact on COVID-19.

## 2. Viral Infections and Immunometabolic Disease

Like other diseases associated with the coronavirus family such as SARS, COVID-19 is mainly a disease of the respiratory system that could interact with the metabolic and endocrine system [13]. Enteroviruses [14], hepatitis C virus [15] and prion-like protein aggregates [16] have been suspected to play a role in the pathobiology of diabetes. Diabetics also present defects in adaptive immunity, delayed Th-1 type hypersensitive reactions and reduced lymphocyte proliferation [11,17]. Defects of the innate response in diabetes come with dysfunction of granulocytes, monocyte/macrophages, dendritic cells, natural killer (NK) cells, B cells, T cells and cytokine signaling [18,19]. Having a parent with diabetes increases the risk of developing diabetes mellitus by 30%–40% [20]. Diabetes-predisposing genes also play important roles in immunity. Individuals carrying these genes are prone to have defective immune defenses [12]. Studies in rodents have shown that after MERS-CoV infection, T2D mice presented immunoinflammatory abnormalities and developed an altered profile of cytokines, with increased expression of IL-17α following infection, observing that the presence of both T2D and the viral infection resulted in more deleterious lung damage and a dysregulated immune response [21]. Allard et al. reported the presence of diabetes in 22 patients from 239 with confirmed influenza H1N1 and their odds ratio for ICU admission was 4.29 among hospitalized patients with diabetes compared to those without. They concluded that diabetes triples the risk of hospitalization H1N1 infection and quadruples the risk of ICU admission once hospitalized [22]. A recent study found that among 1122 patients in 88 U.S. hospitals, 570 patients who died or were discharged, the mortality rate was 28.8% in 184 diabetes and/or uncontrolled hyperglycemia patients, compared with 6.2% of 386 patients without diabetes or hyperglycemia. Among the 184 patients with diabetes and/or hyperglycemia who died or were discharged, 40 of 96 uncontrolled hyperglycemia patients (41.7%) died compared with 13 of 88 diabetes patients (14.8%). Uncontrolled hyperglycemia was defined as ≥2 blood glucoses (BGs) >180 mg/dL within any 24-hour period [23]. Direct damage to β-cell islets has been reported when the SARS coronavirus binds to pancreatic ACE2 receptors, reducing insulin secretion and causing β-cell dysfunction and hyperglycemia. Given that T2D increases the expression of angiotensin-converting enzyme in lung, liver and heart, this direct damage to other key organs could explain to a certain extent the findings of multi-organ failure in SARS-CoV severe infections [24]. The report of higher mortality in uncontrolled hyperglycemia patients reported by Bode et al. [23] and the fact that SARS coronavirus binds to pancreatic ACE2 receptors, decreases insulin secretion and causes b-cell dysfunction [24] lead to a valid speculation that uncontrolled hyperglycemia above 180 mg/dL in COVID-19 patients could be a marker of a very severe state.

The genetic basis of type 1 diabetes (T1D) is well established, with more than 60 identified genes explaining 80% of its heritability. Predisposing gene variants to T1D such as *INS*, *PTPN22*, *IL27* and *IFIH1* involved in immune function, regulation of T-cell activation or innate virus immunity have already been characterized [25,26]. Additionally, hyperglycemia is linked with both chronic inflammatory processes and diabetes related vulnerability to infection. It affects innate immunity by impeding the production of type I interferon, which has multiple effects, including antiviral activity. Peripheral blood mononuclear cells show an impaired production of IL1β, a key mediator in inflammation in diabetics, indicating reduced innate cell activation [12]. sCD40L is shed by activated T lymphocytes and platelets. Plasma levels of sCD40L are elevated in hyperglycemic T2D patients. Immune activation is achieved by binding of CD40L to T cells, macrophages or B cells. Hyperactivation of CD40 bolsters the production of proinflammatory cytokines and the inflammatory milieu, downregulating antigen-specific responses [27]. IL15 is a membrane-associated molecule that promotes the activation of NK and CD8 T-effector memory cells. Expression of IL15/IL15Ra occurs in viral infections. Pathogenic elevated serum levels of IL15 have been reported in T1D patients [9,28]. A recent study demonstrated that O-GlcNAc transferase (OGT), a key enzyme for protein O-GlcNAcylation, mediated influenza A virus (IAV)-induced cytokine storm. The hexosamine biosynthesis pathway (HBP)-associated O-GlcNAc enzyme OGT was induced by IAV to bind to interferon regulatory factor–5 (IRF5). O-GlcNAcylation of IRF5 is required for ubiquitination of IRF5 and subsequent cytokine production. They identified a molecular mechanism by which HBP-mediated O-GlcNAcylation regulates IRF5 function during IAV infection, highlighting the importance of glucose metabolism in IAV-induced cytokine hyperinflammatory responses [29]. This evidence clearly demonstrates that diabetics have dysfunctional innate and adaptive immune responses contributing to an increased susceptibility to viral, bacterial and fungal infections. The abnormal diabetic pathophysiology alters leukocyte normal activities such as chemotaxis, phagocytosis and the ability to kill intracellular pathogens [30]. 

## 3. At the Crossroad of COVID-19 and Diabetes Epidemiology

Several family-based studies of disease heritability have indicated that type 2 diabetes (T2D) is strongly heritable and highly prevalent in large extended families where one or two members are diagnosed with the disease, and this heritability is on average 25% [31]. According to the International Diabetes Federation, diabetes caused 4.2 million deaths in 2019. There are 463 million adults with diabetes in the world. By 2045 this will rise to 700 million. 1.1 million are children and adolescents with type 1 diabetes. Reports from the WHO as of May 15th, 2020 indicated that the SARS-Cov-2 virus has resulted in more than 4,700,000 confirmed infections and 315,000 deaths worldwide. In the US, reports indicate more than 1,450,000 confirmed infections and 89,000 deaths. The CDC suggested that between 160 million and 210 million Americans could contract the disease over a 12-month period. Based on mortality data and current hospital capacity, the number of deaths under the CDC’s scenarios could range from 200,000 to 1.7 million [32]. Very early reports from January 2020 regarding the COVID-19-Diabetes connection should have alarmed the field when, out of 41 confirmed COVID-19 patients admitted to the hospital in China, eight were diabetic (20%), 13 (32%) patients were admitted to an ICU and six (15%) died [33]. Yang and colleagues reported that diabetes was found in 22% of 32 non-survivors from a group of 52 intensive care unit patients with novel COVID-19 [9]. Zhang and colleagues showed that of 140 patients who were admitted to the hospital with COVID-19, 12% had diabetes [34]. Another study reported 16.2% of diabetes among 173 patients with severe disease out of 1099 patients with confirmed COVID-19 [35]. 

Guo et al. reported a mortality rate from COVID-19 infected patients among people with diabetes and without other comorbidities of about 16%. This paper also highlighted that there could be an initial, milder evolution and symptoms of the SARS-CoV-2 infection in diabetic individuals with a consequent delay in appropriate and aggressive intervention that may lead to catastrophic and life-threatening late outcomes. Interleukin-6 (IL-6), fibrinogen and C-reactive protein were reported significantly more elevated in the patients with diabetes and COVID-19 infection [8]. A recent meta-analysis including 1,576 infected patients from seven studies reported a prevalence of diabetes of 9.7% (95% CI: 7.2%–12.2%) [36]. The global prevalence of diabetes among adults over 18 years of age rose from 4.7% in 1980 to 8.5% in 2014 according to WHO data [37]. Reports from the Chinese Centre for Disease Control and Prevention from 72,314 cases of COVID-19 showed that patients with diabetes had a threefold higher mortality rate than did those without diabetes (7.3% vs. 2.3%, respectively). This is perhaps the largest published study relevant to COVID-19 patients and diabetes [38]. Although we all understand that this is a new lethal disease and the information is urgently needed, caution is advisable when interpreting the findings from these studies. Most of the data presented is mainly cross-sectional, not longitudinal comparing follow-up data over time, and the number of subjects most of the time is small, without a control group. Although diabetes has been found highly prevalent among severely COVID-19 affected patients in most of these studies, it is interesting to note that, in Europe for example, nearly all coronavirus deaths are people aged 60 or older, and many of these patients are diabetic.

## 4. Obesity Meets COVID-19

Obese people are up to 80 times more likely to develop T2D [39]. Researchers from France recently reported that obesity (BMI >30 kg/m^2^) and severe obesity (BMI >35 kg/m^2^) were present in 47.6% and 28.2% from 124 consecutive patients admitted in intensive care single center for SARS-CoV-2. They concluded that COVID-19 disease severity increases with higher BMI [40]. Researchers from NYU Langone Health reported that out of the 3615 individuals who tested positive for COVID-19, 775 (21%) had a body mass index (BMI) 30–34, and 595 (16% of the total cohort) had a BMI >35. There were 1853 (51%) patients discharged, 1331 (37%) were admitted to the hospital in acute care and 431 (12%) were either directly admitted or transferred to the ICU during admission. They concluded that although patients aged <60 years are generally considered a lower risk group of COVID-19 disease severity, based on their data, obesity in this age group appears to be a previously unrecognized risk factor for hospital admission and need for critical care [41]. A recent editorial also highlighted that the increased prevalence of obesity in older adults in Italy compared with China may have been a key factor regarding the differences in mortality between the two countries [42]. The authors mentioned that from the millions of people infected with the 2009 H1N1 influenza virus, thousands were hospitalized and unfortunately died. Obesity and severe obesity were identified as main risk factors for hospitalization and mechanical ventilation [43]. They particularly mentioned the case of California where out of 1088 H1N1 influenza virus patients hospitalized in critical condition, 268 were older than 20 years old from whom 156 cases (58%) had a BMI ≥ 30. Of these, 67 (43%) had a BMI ≥ 40 [44]. This was also the 2009 H1N1 story for New Mexico where 46% had obesity, and 56% of those requiring mechanical ventilation had severe obesity [45].

## 5. Mirror Images: Immunoinflammatory Pathobiology of COVID-19 and Diabetes

Yang et al. also reported in their meta-analysis that compared the non-intensive care unit (ICU) vs. ICU COVID-19 patients, ICU patients had higher plasma levels of proinflammatory cytokines IL2, IL7, IL10, GSCF, IP10, MCP1, MIP1A and tumor necrosis factor (TNFα) [36]. As in these COVID-19 infections, there is evidence supporting the involvement of inflammation in the pathogenesis of both type 1 (T1D) and T2D. Mechanisms thought to be responsible for the inflammatory state in T2D include activation of the nuclear factor-κB (NF-κB) and JUN N-terminal kinase (JNK) pathways, activation of interleukin-1β (IL-1β), IL-6, TNFα and recruitment and activation of immune cells [12]. Diabetes and hypertension share common pathways among them the sympathetic nervous system (SNS), the renin-angiotensin-aldosterone system (RAAS) including angiotensin-converting enzyme (ACE), oxidative stress, adipokines, insulin resistance (IR) (currently considered a prothrombotic state) and peroxisome proliferator-activated receptors (PPARs) [46]. The SARS-CoV-2 envelope spike glycoprotein binds to the angiotensin-converting enzyme 2 (ACE2) to access human cells. ACE2 degrades angiotensin II into angiotensin 1–7. ACE2 inhibition allows angiotensin II to bind angiotensin 1 receptor (AT1R) or AT2R to exert proinflammatory responses. Insulin resistant and T2D patients with severe COVID-19 infection may have an increased activation of AT1R/AT2R [47]. We have been describing how diabetic patients would be at higher risk for fatality than non-diabetic if COVID-19 infection is present. However, we should also consider that non-diabetic COVID-infected individuals could be at a higher risk as well to develop diabetes as a long-term consequence of this infection. ACE2, the access of SARS-CoV-2 to enter human cells, is fully expressed in hepatocytes and β-cells with the expected infection of liver and pancreas during an acute infection [48]. Therefore, a potential impairment of liver glucose utilization and uptake and pancreatic insulin secretion could be a devastating acute and/or long consequence of COVID-19 infection in some individuals. SARS-CoV-2 could also predispose the immune system of certain individuals to overreact against their β-cells and develop autoimmune diabetic disease. Therefore, we must always keep in mind that there could be a dark side of COVID-19 towards the insulin–glucose axis given its role in acutely infecting the liver and β-cells at a molecular level.

Diabetes is the hallmark of immunometabolic diseases, a cluster of chronic pathologies, including insulin resistance, hypertension, dyslipidemias and cardiovascular disease (CVD). They are the leading cause of death across the world. These pathologies are associated with multiple immune risk factors of metabolic origin that may not be obvious in symptom-free individuals [49,50]. Recent research has increased our awareness of the key role that adipose tissue (AT) dysfunction plays in the development of IR, the underlying cause of T2D and other immunometabolic diseases [51]. The core mechanisms of AT dysfunction involves localized immunometabolic processes during AT expansion involving the immune response, such as impaired angiogenesis and hypoxia, inflammation, inappropriate extracellular matrix (ECM) remodeling and fibrosis, that would inexorably become systemic at later stages. AT dysfunction is characterized by macrophage infiltration into adipose tissue [51]. Interactions between macrophages and adipocytes represent the early molecular events that will lead to subclinical inflammation, ultimately giving rise to IR and its sequelae (glucotoxicity, lipotoxicity, endothelial dysfunction, systemic inflammation and CVD) [49,52]. There is a current lack of clinically oriented indicators to assess the complex phenomenon of AT dysfunction for early detection of cardiovascular and immunometabolic risk before it develops into an evident systemic (muscle and liver) IR, overt metabolic syndrome and chronic subclinical low-grade systemic inflammation [49,53] (Figure 1).

## 6. Chronic Low-Grade Subclinical Systemic Inflammation in Prediabetes and Adipose Tissue Dysfunction Meets COVID-19

An adiponectin/leptin ratio (ALR) has been recently developed as a functional marker of adipose tissue (AT) dysfunction [54]. Individuals with a low ALR are characterized by a lower secretion of antiinflammatory adiponectin in relation to proinflammatory leptin levels, unhealthy adipose tissue hypoxia, proinflammatory macrophage polarization, altered adipokine profile and IR. These individuals are at higher risk for systemic subclinical inflammation, cardiovascular and immunometabolic pathologies [53,55]. A consequence of chronic positive energy balance leading to AT dysfunction is an ectopic deposition of non-esterified fatty acids (NEFA) as triacylglycerol in the liver, skeletal muscle and pancreas promoting lipotoxicity, a proinflammatory state and IR [56]. Another new biomarker already validated, the adipose tissue-insulin resistance (IRi) index (Adipo-IR**_i_** = plasma-free fatty acids × fasting plasma insulin (FPI; mmol/L/pmol/L)) is calculated based on the linear relationship between the rise in the FPI level and inhibition of the rate of fasting plasma NEFA [57]. Individuals with an elevated Adipo-IR**_i_** have a more pronounced adipose tissue IR leading to a more severe local and systemic proinflammatory state [58]. Wen et al. reported that the degree of adipose tissue insulin resistance increases in obesity and T2D, and is associated with prediabetes [59]. Recent published research from Rodriguez et al. reported data from systemic measurements of the ALR and the Adipo-IRi index in symptom-free adults to characterize AT dysfunction [49]. The investigators used age, gender, weight, waist and % body fat matched groups of volunteer study subjects. Their blood pressure and biochemical phenotypic measurements for fasting plasma glucose and triglycerides were within normal ranges. A pattern of metabolically driven inflammation characterized by increased circulating leptin concentrations along with a decreased levels of adiponectin indicative of an impaired adipose tissue adipokinome was found in the symptom-free subgroup with a low (L) ALR. Proinflammatory fasting systemic biomarker levels between the high (H) and low (L) ALR and Adipo-IRi groups showed higher systemic levels of TNF-α, IL-6, hs-C-reactive protein (CRP) and PAI-1 in the symptom-free subjects with (L) ALR. Their data also showed that the mean homeostasis model assessment (HOMA) was frankly elevated and the insulin and glucagon-like peptide 1 (GLP-1) postprandial curves and their area under the curve (AUC) were increased in the (L) ALR group compared to the subjects with a (H) ALR. Particularly, the insulin curve showed striking elevation in the individuals with (L) ALR. They concluded that in apparently symptom-free individuals a cluster of altered immunometabolic phenotypes related to impaired angiogenesis and hypoxia, inflammation, inappropriate extracellular matrix (ECM) remodeling and macrophage polarization could be place into a systemic and molecular construct coined as adipose tissue dysfunction, which would trigger the early events leading to the development of insulin resistance and systemic inflammation [49].

**Figure 2:**
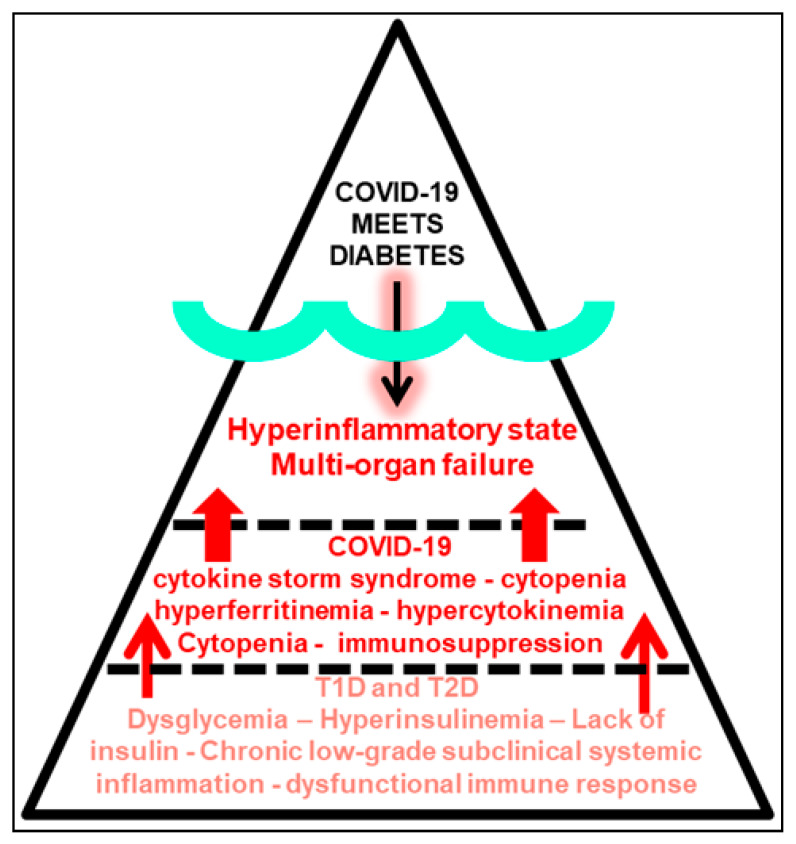
COVID-19 meets diabetes at the tip of the triangle above the surface representing the environment, viral exposure and infection. SARS-CoV-2 enters human cells. Once inside the body (underneath the triangle’s surface) a diabetic state represented by a chronic low-grade subclinical systemic inflammation and a dysfunctional immune response (bottom part of the triangle) may host the scenario to enhance the deleterious cytokine storm hyperinflammatory state after COVID-19 infection (center of the triangle) leading to severe multi-organ failure. The COVID-19 pandemic mirrors the diabetes pandemic, or vice versa.

This deleterious scenario could also have somehow served as the underlying mechanism enhancing a COVID-19-driven cytokine storm-like hyperinflammatory state in subjects without overt diabetes but with undetected immunometabolic abnormalities and chronic low-grade systemic inflammation, which when severely infected with COVID-19, trigger multi-organ failure and death [60]. Therefore, a great deal of individuals exposed to COVID-19, but with subclinical inflammation, adipose tissue dysfunction and prediabetes might also be at a higher risk of developing severe life-threatening respiratory illness after COVID-19 infection (Figure 1 and Figure 2). There is accumulating evidence suggesting that a subgroup of patients with severe COVID-19 might have profound myocarditis, fatal arrhythmias [36], and brainstem infection leading to respiratory failure and encephalopathy, and a cytokine-like storm syndrome with hyperinflammation (liver, heart and kidney), cytopenias, hyperferritinemia, severe hypercytokinemia and multiorgan failure (Figure 2) [61]. A recent preprint from Petrilli et al. reported a cross-sectional analysis of all patients with laboratory-confirmed COVID-19 infection treated at an academic health system in New York City between 1 March 2020 and 2 April 2020. Among 4103 COVID-19 patients, 1999 were hospitalized, of whom 292 (14.6%) died. 445 patients required mechanical ventilation, of whom 162 (36.4%) died. The most relevant hospitalization risk criteria were age 65–74 years, BMI >40 and heart failure. A key question would be: how many of these obese individuals were metabolically unhealthy? [62]. For critical illness, the most important parameters were abnormal oxygen saturation, d-dimer, ferritin, procalcitonin and the proinflammatory C-reactive protein (CRP) >200. The two more important features in the decision tree for admission were age >65 and obesity [63]. Of note, this new research document has not been yet certified by peer review; therefore it should not be used to guide clinical practice.

A valid question is why some COVID-19 positive-test cases present with only mild symptoms compared to others that have severe symptomatology ending up with fatal outcomes. So far, all data indicates that people who are older, immunocompromised or have significant underlying conditions such as heart failure, coronary heart disease, arrhythmia, diabetic nephropathy and mainly any kind of limited lung function, asthma, acute or chronic pulmonary disease among others, are more likely to develop poorer outcomes and multiorgan failure [61]. However, it is unclear why COVID-19 has caused the death of perhaps thousands of young and not so young people without any apparent underlying health conditions. Scientists are desperately trying to unravel why COVID-19 can be lethal to young and apparently healthy individuals. They speculate that genetics play a key role, and some young people might have structural variations in the gene that encodes the ACE2 receptor influencing its binding with the SARS-CoV-2 spike protein, making them more susceptible to COVID-19 infection [64]. SARS-CoV-2 supposedly affects women less than men due to the presence of two X chromosomes in women, with immune regulatory genes encoded by X chromosome in female gender causing lower viral load levels [65]. Indeed, current data shows that the overall COVID-19 fatality rates are much higher for men. Some other factors indicate that perhaps men develop a higher susceptibility than women due to unhealthy lifestyle habits such as cigarette smoking and alcohol consumption as examples, contributing to lung and liver disease, which are risk factors for severe COVID-19 according to the CDC [66,67]. The severity of some cases in the young may also be related to the viral load at the time of exposure to the virus [6].

According to the WHO, 34% of men over the age of 15 are cigarette smokers compared to 6% of women. 45% of the US population over the age of 50 is obese. The US Department of Health and Human Services has reported that African Americans are also dying from the virus at alarmingly disproportionate rates. The risk of a severe coronavirus outcome is increased by high blood pressure and diabetes. These are conditions more prevalent among black Americans. Nearly a third of African American adults have high blood pressure, compared to 24% of white adults. Unhealthy life styles, cigarette smoking, alcohol consumption, obesity, diabetes, high blood pressure and male gender are clusters of cardiometabolic risk factors always accompanying the presence of the metabolic syndrome [68]. We are well aware now that major inflammatory mechanisms contribute to the pathogenesis and progression of this syndrome [69]. Adipose tissue (AT) dysfunction, monocytes, macrophages and phagocyte activity contribute to the proinflammatory milieu with the release of leptin, low adiponectin, chemokines, cytokines, circulating inflammatory CRP and fibrinogen promoting systemic inflammation and insulin resistance. Therefore, by far, the metabolic syndrome is an inflammatory disorder [69]. The combined data from the reports of Rodriguez et al. [49] and Petrilli et al. [63] seem to indicate, perhaps, that most symptom-free obese individuals with severe and fatal COVID-19 could be metabolically unhealthy [62], or more concerning, that non-obese symptom-free individuals with underlying but undetected adipose tissue dysfunction and a low ALR with a high Adipo-IRi index exposed to COVID-19 infection are also at high risk for severe and fatal COVID-19 outcomes [49]. Metabolically unhealthy obese and individuals with adipose tissue dysfunction present key proinflammatory conditions that are highly underestimated and rarely diagnosed among symptom-free individuals (Figure 1). The systemic proinflammatory setting in symptom-free individuals without overt diabetes or hypertension but with metabolically unhealthy obesity or non-obese adipose tissue dysfunction could require hospitalization and progress to a more severe and critical reaction requiring ICU intervention. In turn, this could perhaps contribute to enhance the hyperinflammatory state in symptom-free individuals infected with severe COVID-19 triggering multi-organ failure as shown in Figure 2.

## 7. Conclusions

We brought attention to two manifestations of a highly complex, multifactorial, developmental and environmentally dependent pathology of critical importance to human survival [70]. Although very different in their presentation and transmission, the acute communicable COVID-19 pandemic mirrors the chronic non-communicable diabetes pandemic in many pathobiological aspects. Our interest was to emphasize the ties between the intimate molecular immunoinflammatory mechanisms that relate and enhance the morbidity and lethality when COVID-19 meets diabetes (Figure 2). We should focus on how to use all information and scientific data to address both pathologies and study their common underlying pathways. For example, recent publications highlighted the significance of the two coronavirus receptor proteins, angiotensin converting enzyme 2 (ACE2) and dipeptidyl peptidase-4 (DPP4), also highlighting their dual physiologic effects as transducers of metabolic signals and pathways regulating inflammation, renal and cardiovascular physiology and glucose homeostasis [71]. This opens the door to consider glucose-lowering agents such as the DPP4 inhibitors as tools to intervene in the interaction of COVD-19 and dysglycemic states [72]. 

There is still much to learn. Important considerations should include if the natural history of the COVID-19 infection is different between T1D and T2D individuals, how age relates to disease outcomes and severity among patients with or without diabetes, and whether antidiabetic medications affect disease progression in infected diabetic individuals. Given the compelling data related to the two disorders, we should not be surprised by the more serious consequences for those with frank diabetes or a dysglycemic state [73,74] infected with COVID-19. Although it is not surprising, this relationship is however frightening given the fast transmission rate of SARS-CoV-2 and the global prevalence of diabetes. This unfortunate scenario should motivate the research community to use the current situation, though dire, to make meaningful progress in preventing and treating both epidemics.
